# Vascular bone tumors of the pelvis and extremities: an 18-case clinical and radiological analysis

**DOI:** 10.1007/s00402-026-06207-5

**Published:** 2026-02-27

**Authors:** Recep Öztürk, Fisun Ardıç Yükrük

**Affiliations:** 1 Department of Orthopedics and Traumatology, Ankara Onkoloji Eğitim ve Araştırma Hastanesi, Ankara, Turkey; 2 Department of Pathology, Ankara Onkoloji Eğitim ve Araştırma Hastanesi, Ankara, Turkey

**Keywords:** Hemangioma, Angiosarcoma, Epithelioid hemangioendothelioma, Radiology

## Abstract

**Background:**

Primary vascular bone tumors are rare, spanning from benign to highly malignant lesions. Pelvic and extremity involvement is uncommon, and differentiation between benign and malignant tumors remains challenging due to overlapping radiological and pathological features. This study aimed to identify imaging predictors of malignancy and evaluate clinical outcomes in vascular bone tumors of the pelvis and extremities.

**Methods:**

We retrospectively analyzed 18 patients diagnosed with vascular bone tumors between 2013 and 2024. Tumors were classified as benign (hemangioma, epithelioid hemangioma; *n* = 11) or malignant (angiosarcoma, epithelioid hemangioendothelioma; *n* = 7). Demographic, radiological (plain radiographs, MRI, CT), and clinical data were collected. Cortical expansion/destruction, pathological fractures, soft tissue components, and tumor size were assessed. Statistical analysis included Fisher’s exact test, Mann–Whitney U test, Spearman correlation, and ROC analysis to identify predictors of malignancy.

**Results:**

Median age was 41.0 years for benign and 53.0 years for malignant tumors. Soft tissue components were significantly more frequent in malignant tumors (57% vs. 9%, *p* = 0.025), and tumor size was larger (mean 9.0 cm vs. 3.7 cm, *p* = 0.001). Cortical expansion, destruction, and pathological fractures did not differ significantly. ROC analysis suggested that larger tumor size (≥ 5.5 cm in this cohort) was associated with malignancy with 85.7% sensitivity and 90.9% specificity (AUC = 0.929, *p* = 0.003) and should be interpreted as an exploratory finding. All benign tumors underwent intralesional curettage, with no recurrences or complications observed. In contrast, malignant tumors exhibited high rates of relapse and mortality, with only one patient with epithelioid hemangioendothelioma surviving with stable disease at 112 months.

**Conclusion:**

In vascular bone tumors of the pelvis and extremities, the presence of a soft tissue component and tumor size ≥ 5.5 cm are among the most useful radiological features associated with malignancy. While benign lesions generally have excellent outcomes with curettage, malignant tumors are associated with a poor prognosis. Histopathological confirmation remains essential, and larger series are needed to refine diagnostic and prognostic criteria.

## Introduction

Primary vascular tumors of bone are exceedingly rare entities [1]. They most commonly occur in the skull and vertebral column, whereas involvement of other skeletal regions is uncommon [2]. These tumors encompass a wide histopathological spectrum ranging from entirely benign lesions to intermediate forms and highly aggressive malignant neoplasms [1]. Although several classification systems have been proposed, a universally accepted scheme is still lacking [3], mainly due to inconsistent terminology, the absence of well-defined histological criteria, and limited clinicopathological correlation [3].

Currently, intraosseous hemangioma is recognized as a benign vascular tumor of bone, whereas angiosarcoma represents the high-grade malignant end of the spectrum. Epithelioid hemangioma has recently been better defined and, despite its benign classification, may behave locally aggressively and extend into soft tissues, positioning it within the intermediate category [4]. Epithelioid hemangioendothelioma, on the other hand, is considered a low-grade malignant vascular tumor [5]. More recently described pseudomyogenic hemangioendothelioma represents another intermediate vascular tumor, typically showing an indolent clinical course with minimal risk of distant spread [6]. Despite these defined entities, data regarding vascular tumors arising specifically in rare locations such as the pelvis and extremities remain limited.

The management of primary vascular bone tumors is challenging due to their broad biological behavior, lack of specific clinical or radiological features, difficulty in early diagnosis, and—perhaps most importantly—the often limited correlation between histopathological appearance and clinical course [2, 6]. This study aims to analyze the demographic characteristics, radiological features, and clinical outcomes of vascular bone tumors located in the pelvis and extremities, to identify imaging clues that may raise suspicion for vascular tumors, and to determine the factors predictive of malignancy. Additionally, clinicopathological correlation among these rare tumors was evaluated.

## Methods

This retrospective study included patients diagnosed with vascular bone tumors of the pelvis or extremities based on histopathological evaluation who were treated and followed in our orthopaedics department between 2013 and 2024. Patients without histopathological confirmation, as well as asymptomatic individuals followed solely with clinical or radiological examinations, were excluded. Demographic information, radiological findings obtained from plain radiographs, MRI, and when available, CT, whole-body bone scintigraphy, or PET imaging, histopathological reports, tumor characteristics including anatomical location and gross size, surgical procedures, perioperative chemotherapy or radiotherapy administered to malignant cases, follow-up duration, and clinical outcomes were extracted from the hospital information system, patient files, and operative records. This study was approved by the Institutional Review Board (Approval No: 2021-01/965).

In accordance with contemporary classifications, vascular tumors of bone may be categorized as hemangioma (benign), epithelioid hemangioma (intermediate, locally aggressive), pseudomyogenic hemangioendothelioma (intermediate, rarely metastasizing), and angiosarcoma or epithelioid hemangioendothelioma (malignant) [1]. For this study, cases were grouped into two categories: benign tumors, including hemangioma and epithelioid hemangioma, and malignant tumors, comprising angiosarcoma and epithelioid hemangioendothelioma. No cases of pseudomyogenic hemangioendothelioma were identified during the study period.

All imaging studies were reviewed by two fellowship-trained musculoskeletal radiologists with more than 10 years of experience. Experienced musculoskeletal pathologists established histopathological diagnoses, and difficult cases were discussed in a multidisciplinary tumor board to reach consensus.

### Statistical analysis

Statistical analyses were performed using SPSS software (version 27.0; SPSS Inc., Armonk, NY). The distribution of continuous variables was assessed using the Kolmogorov–Smirnov and Shapiro–Wilk tests. Normally distributed variables were presented as mean and standard deviation, and non-normally distributed variables as median and interquartile range. Categorical variables were summarized as frequencies and percentages. Comparisons of proportions between groups were made using Fisher’s exact test and likelihood ratio tests due to the small sample size, and the Mann–Whitney U test was applied for comparisons of numerical variables. Receiver operating characteristic (ROC) analysis was conducted to identify an exploratory tumor size threshold associated with malignancy. Statistical significance was defined as a p-value < 0.05.

## Results

A total of 18 patients were included in the study (Table 1). The benign tumor group comprised 11 patients, including 9 cases of intraosseous hemangioma and 2 cases of epithelioid hemangioma, while the malignant tumor group included 7 patients, consisting of 6 cases of angiosarcoma and 1 case of epithelioid hemangioendothelioma. The mean age was 41.5 years in the benign group and 52.0 years in the malignant group. In the benign group, lesions were most frequently located in the femur, followed by the tibia, humerus, and small bones of the hand and foot. In the malignant group, lesions were mainly located in the femur and pelvis.

**Table 1:** Patient characteristics, tumor localization, treatment, and follow-up

No	Age/Sex	Localization	Diagnosis / Grade	Treatment	Follow-up (Months)	Complications	Last status
1	25/M	2nd Metacarp	Hemangioma	Curettage + Graft	13	–	NA
2	60/F	Ilium	Hemangioma	Curettage + RT	12	–	NA
3	55/F	Medial Malleolus	Hemangioma	Curettage + Graft	121	–	NED
4	35/F	Tibial diaphysis	Hemangioma	Curettage + Graft	40	–	NED
5	11/M	Distal Femur	Hemangioma	Curettage + Graft	85	–	NED
6	8/F	Humerus diaphysis	Hemangioma	Curettage + Graft	81	–	NED
7	72/M	Proximal Femur	Hemangioma, Epithelioid	Curettage + Cement + IF	18	–	NED
8	39/M	2nd Metacarpal	Hemangioma	Curettage + Graft + IF	15	–	NED
9	51/M	Tibial diaphysis	Hemangioma	Curettage + Graft + IF	12	–	NED
10	41/F	Proximal Femur	Hemangioma	Curettage + Cement + IF	14	–	NED
11	60/F	Proximal Femur	Hemangioma, Epithelioid	Curettage + Cement + IF	13	–	NED
12	56/M	Pelvis (Ilium to Sacrum, Inferior Ischium)	Angiosarcoma, G3	Wide Resection + Allograft	6	Deep infection, local recurrence	DOD
13	53/M	Distal Femur	Angiosarcoma, G3	Wide Resection + Distal Femur Prosthesis	5	Relapse	DOD
14	36/M	Subtrochanteric Femur	Angiosarcoma, G3	Wide Resection + Tumor Prosthesis + CT	16	Lung Metastasis	DOD
15	54/M	Proximal Femur	Epithelioid Hemangioendothelioma	Wide Resection + Proximal Femur Prosthesis	112	Lung Metastasis	AWD
16	46/F	Multifocal (Pelvis, Proximal Femur, Humerus, Ribs, Thoracic & Lumbar Spine)	Angiosarcoma	Wide Resection + RT + CT	11	Progression	DOD
17	29/F	Distal Femur	Angiosarcoma, Epithelioid	Wide Resection + Distal Femur Prosthesis + RT	12	Pulmonary embolism	DOC
18	76/M	Pelvis (Ilium to Sacrum)	Angiosarcoma, Epithelioid	Wide Resection + Lumbopelvic Stabilization	5	Deep infection, DVT	DOC

No: Number, M: Male, F: Female, Phal: Phalanx, Med: Medial, Diaph: Diaphysis, Prox: Proximal, Epith: Epithelioid, Hemangioend: Hemangioendothelioma, C: Curettage, Cem: Cementation, G: Grafting, IF: Internal Fixation, RT: Radiotherapy, FU: Follow-up, NA: Not Available, WR: Wide Resection, Allogr: Allograft, CT: Chemotherapy, TP: Tumor Prosthesis, NED: No Evidence of Disease, DOD: Dead of Disease, AWD: Alive with Disease, DVT: Deep Vein Thrombosis

**Fig. 1:**
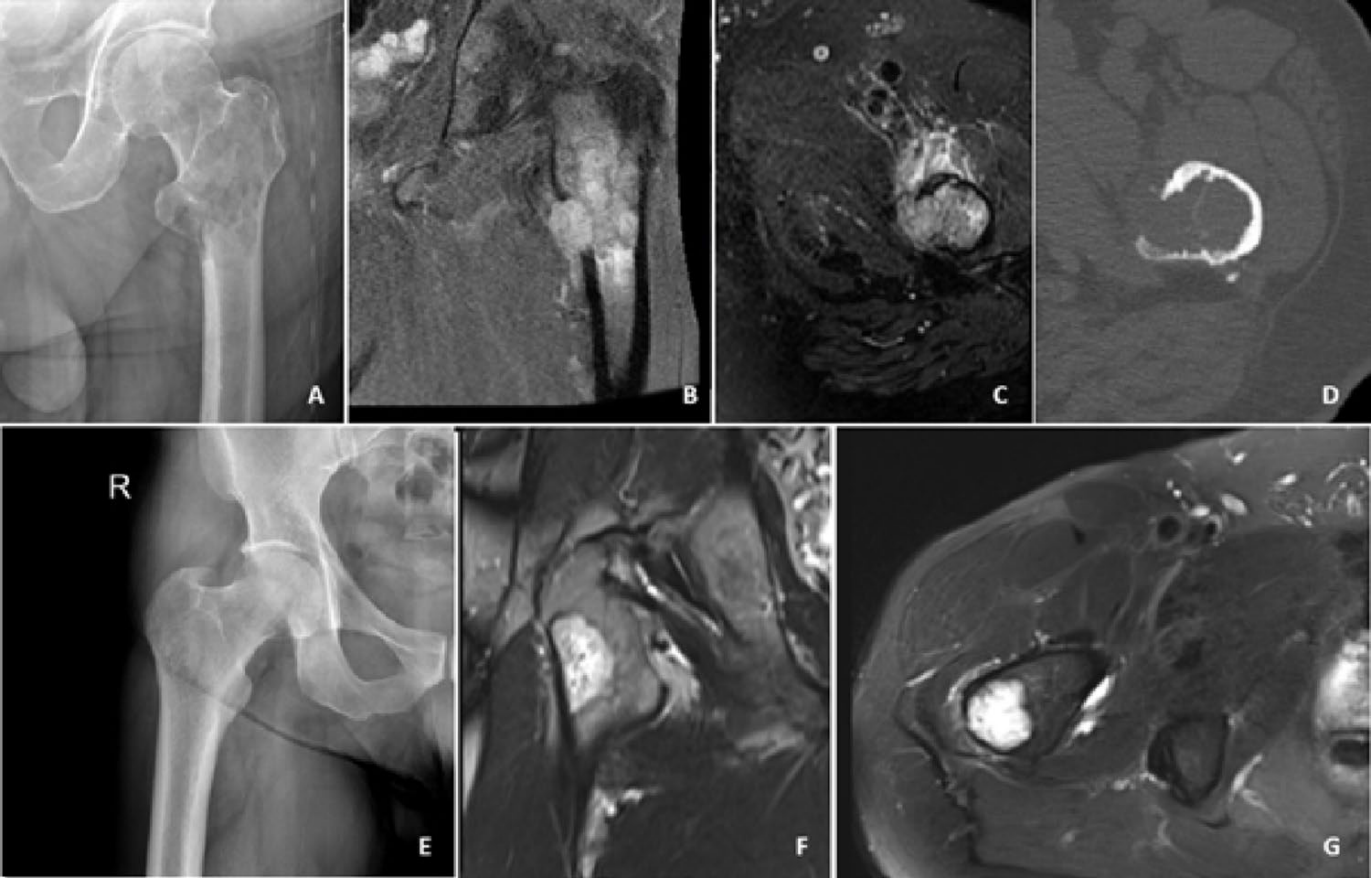
– Benign vascular bone tumors may demonstrate aggressive radiological features overlapping with malignant lesions. A–D) 72-year-old male patient with subtrochanteric epithelioid hemangioma; radiographs, MRI, and CT slices show a pathological fracture and a soft tissue component. E–G) 54-year-old male patient with epithelioid hemangioendothelioma; radiographs and MRI slices show an eccentric medullary lesion without cortical destruction or expansion

**Fig. 2:**
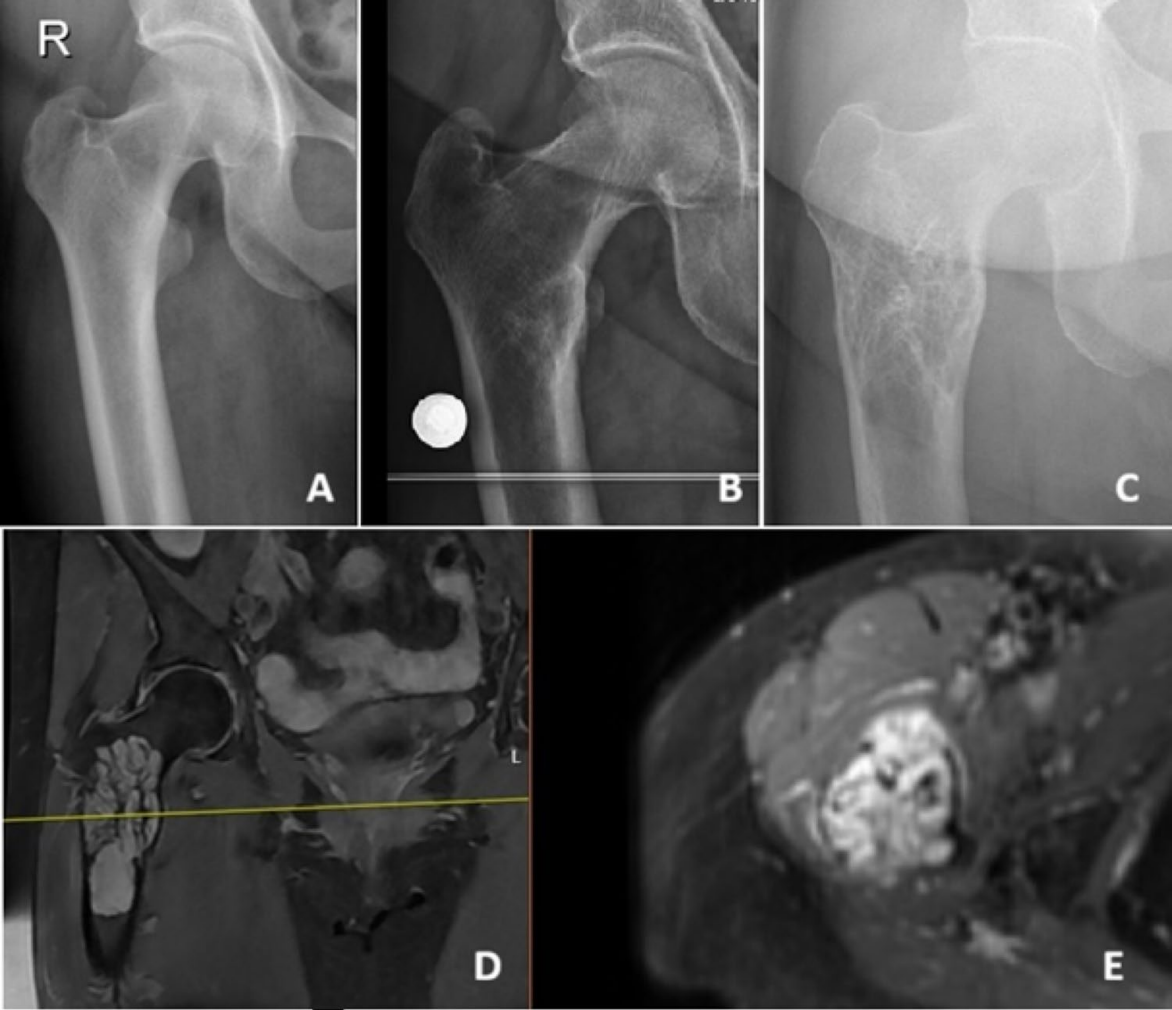
– Epithelioid hemangioma in the proximal femur. (A) Radiograph of the right hip 10 years before presentation, no detectable lesion. (B) Radiograph of the right hip 2 years before presentation, small medullary lesion adjacent to the lesser trochanter. (C) Radiograph at presentation, showing marked lesion growth. (D) Coronal MRI at presentation, demonstrating a medullary lesion filling the intertrochanteric region and extending below the lesser trochanter. (E) Axial MRI slice shows surrounding reactive changes

**Fig. 3:**
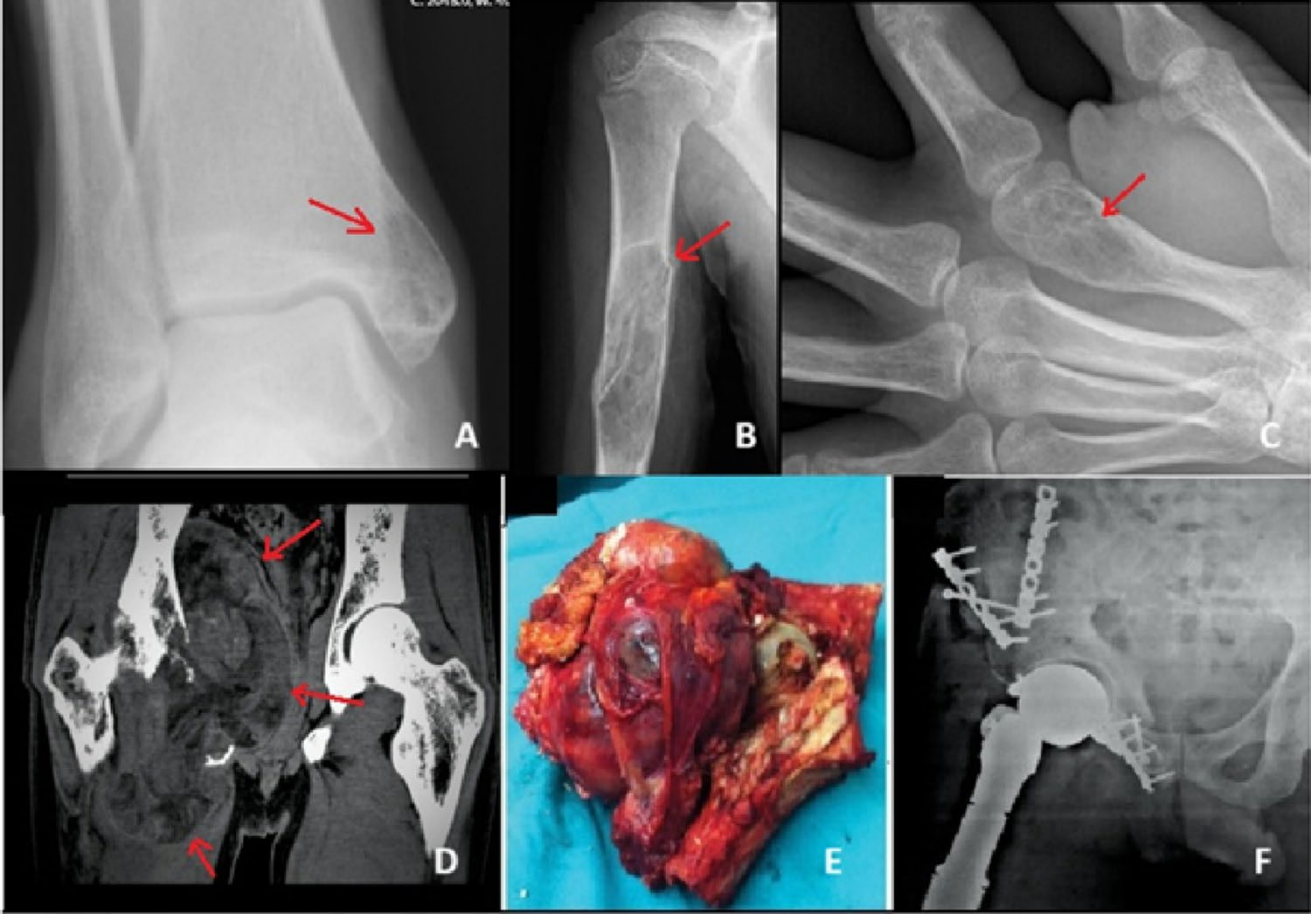
– Examples of vascular bone tumors: (A) Hemangioma in the medial malleolus. (B) Hemangioma in the humeral diaphysis causing a pathological fracture. (C) Hemangioma in the distal metacarpal. (D) MRI of a pelvic angiosarcoma. (E) Resected specimen. (F) Radiograph after reconstruction with fresh-frozen hemipelvis. Arrows indicate the tumors

The most common presenting symptoms were pain and swelling. The median symptom duration before diagnosis was 9 months (range: 1–36 months). The median symptom duration was longer in benign tumors (11 months) compared with malignant tumors (5 months). All 18 lesions demonstrated contrast enhancement on imaging. Cortical expansion and destruction were observed in 55% and 71% of cases in the benign and malignant groups, respectively (Fig. 1). Pathological fractures occurred in 9% of benign tumors and 29% of malignant tumors (Fig. 2). Soft tissue components were present in 9% of benign and 57% of malignant tumors (Fig. 3). All benign tumors were primary and localized. Except for one multifocal malignant case, all malignant tumors were localized at diagnosis.

**Fig. 4:**
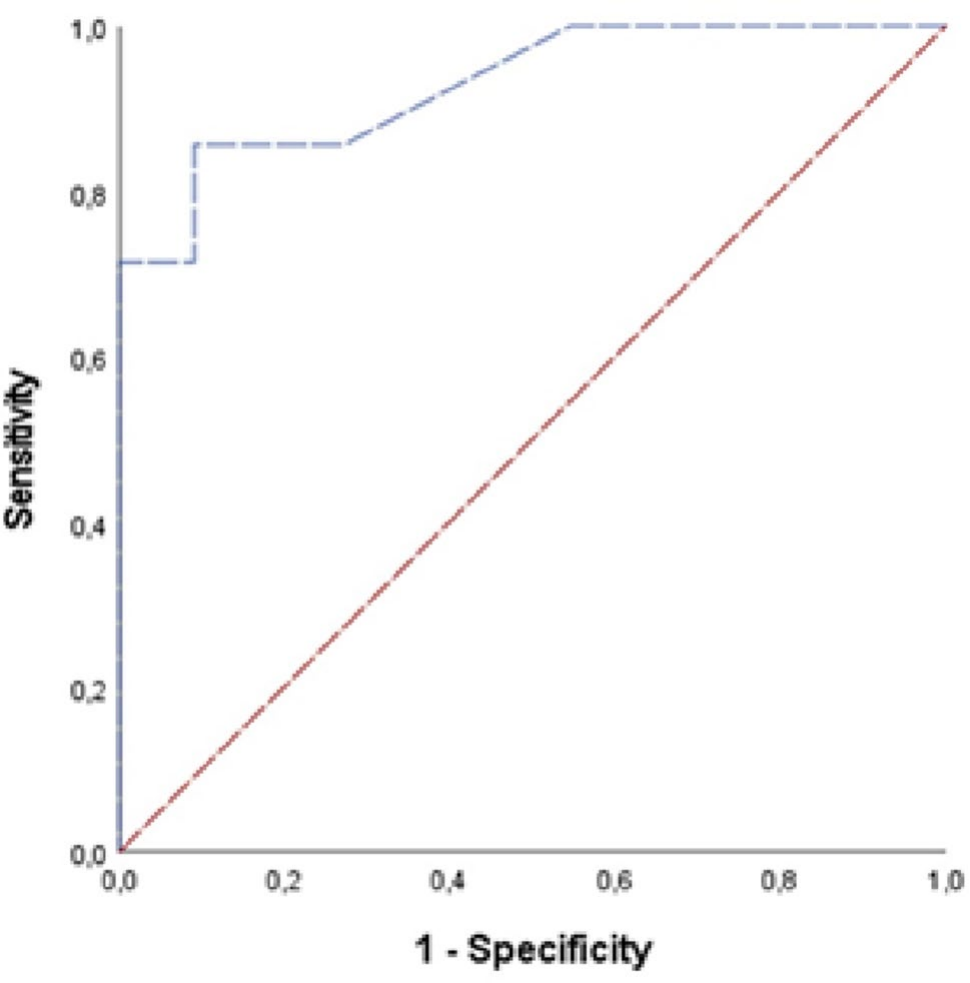
– ROC curve for tumor size to predict malignant tumors. AUC = 0.929, *p* = 0.003

Age, presence of cortical expansion, cortical destruction, pathological fractures, and follow-up duration did not differ significantly between benign and malignant tumors (*p* > 0.05). Soft tissue components were significantly more common (*p* = 0.025), and tumor size was significantly larger in malignant tumors (*p* = 0.001) (Table 2). Spearman correlation analysis revealed a moderate positive correlation between the presence of soft tissue components and malignancy (Rho = 0.523, *p* = 0.026) and a strong positive correlation between tumor size and malignancy (Rho = 0.731, *p* < 0.001). ROC analysis demonstrated that a tumor size exploratory threshold of 5.5 cm was associated with malignancy with 85.7% sensitivity and 90.9% specificity (likelihood ratio 9.42; AUC = 0.929, *p* = 0.003) (Table 3) (Fig. 4).

**Table 2:** Age and imaging findings according to tumor type

Parameter	Benign tumors (*n* = 11)	Malignant tumors (*n* = 7)	*p*-value
Age (median, min–max)	41.0 (8–72)	53.0 (29–76)	0.425*
Cortical expansion, n (%)	6 (54.5)	5 (45.5)	0.637†
Cortical destruction, n (%)	6 (54.5)	5 (45.5)	0.637†
Pathological fracture, n (%)	1 (9.1)	3 (28.6)	0.140†
Contrast enhancement, n (%)	11 (100)	7 (100)	NA
Soft tissue component, n (%)	1 (9.1)	4 (57.1)	0.025‡
Tumor size (cm, mean ± SD)	3.73 ± 1.55	9.00 (IQR 3.37)	0.001*
Follow-up duration (months, median, range)	38.0 (12–121)	23.0 (5–112)	0.126

NA: Not applicable

*Mann-Whitney U test, †Fisher’s exact test, ‡Likelihood ratio

**Table 3:** ROC analysis for tumor size in predicting malignancy

Risk factor	AUC	Std. error	*p*-value	Cut-off (cm)	Sensitivity (%)	Specificity (%)	Likelihood ratio	95% CI
Tumor size	0.929	0.065	0.003	5.50	85.7	90.9	9.42	0.802–1.00

AUC: Area Under the Curve, CI: Confidence Interval

The median follow-up duration was 15.0 months (range, 12–121 months) in the benign group. Except for 2 patients lost to follow-up, all remain disease-free. All 11 benign cases were treated with intralesional curettage. Seven patients underwent grafting, and three had cementation. Internal fixation was applied when required to prevent fracture. No recurrences or complications were observed.

### Clinical course of malignant tumors (Cases 12–18)

#### Case 12

involved a 56-year-old male with a mass extending from the superior acetabulum to the sacrum and inferior ischium, with a soft tissue component compressing the bladder and major vascular structures. The mass extended across the contralateral hemipelvis. The patient underwent wide tumor resection and reconstruction using a fresh-frozen hemipelvic allograft, as radiotherapy was refused. Despite surgery, wide surgical margins could not be achieved. The patient developed deep infection, local recurrence, and pulmonary metastasis, and died 6 months postoperatively (Fig. 3).

#### Case 13

was a 53-year-old male with a distal femur angiosarcoma associated with a soft tissue component (130 × 75 mm), displacing the patella. Wide resection and distal femur tumor prosthesis implantation were performed. Despite adjuvant chemotherapy and radiotherapy, pulmonary metastasis developed due to close surgical margins, and the patient died from a pulmonary embolism 5 months postoperatively.

#### Case 14

involved a 36-year-old male presenting with a subtrochanteric femoral pathological fracture and pulmonary metastasis due to angiosarcoma. Wide resection, tumor prosthesis implantation, and chemotherapy were applied. Metastatic progression occurred at 12 months postoperatively, and the patient died at 16 months.

#### Case 15

was a 54-year-old male with proximal femur epithelioid hemangioendothelioma. Wide resection and proximal femur tumor prosthesis placement were performed. Pulmonary metastases developed; however, after three cycles of chemotherapy, the patient remains under follow-up with stable lesions at 112 months.

#### Case 16

involved a 46-year-old female with multifocal involvement, including the pelvis, proximal left femur, proximal left humerus, ribs, and thoracic and lumbar vertebrae. Corpectomy, decompression, kyphosis correction, and instrumentation were performed, followed by radiotherapy and chemotherapy. She eventually died from disease progression and widespread pulmonary metastases 11 months postoperatively.

#### Case 17

was a 29-year-old female with lipodystrophy and diabetes, presenting with widespread skeletal deformities and a distal left femoral pathological fracture. Wide tumor resection and distal femur prosthesis implantation were performed. Early postoperative pulmonary embolism occurred, leading to patient death.

#### Case 18

involved a 76-year-old male with a mass extending from the ilium to the sacrum. Wide resection with lumbopelvic stabilization was performed. Postoperatively, deep venous thrombosis and deep infection occurred, and the patient subsequently died.

## Discussion

Vascular tumors are among the most confusing entities due to the numerous terms used to describe them. Pathologically, these tumors are strikingly similar, making differentiation challenging. Since the clinical behavior, treatment, and prognosis of vascular bone tumors vary considerably, accurate and effective differentiation is crucial [7, 8]. To our knowledge, this is the first study to compare benign and malignant vascular bone tumors both clinically and radiologically. The most striking findings of the present study were the strong association of tumor size and soft tissue extension with malignancy in vascular bone tumors of the pelvis and extremities. A soft tissue component strongly suggests malignancy, and a larger tumor size should also raise suspicion. In the literature, the proportion of soft tissue component in low-grade epithelioid hemangioendothelioma has been reported as 40% [7]. In angiosarcomas, cortical destruction and invasion into adjacent tissues have been reported in 65% of cases [5, 7, 9]. Specifically, in this cohort, larger tumors (approximately ≥ 5.5 cm) warranted careful evaluation for malignant potential. However, pathological fractures and aggressive radiological features may also be present in benign tumors, indicating that these features alone cannot definitively distinguish malignancy, and histopathological confirmation remains essential [2, 10].

Rigopoulou et al. examined 15 appendicular skeletal hemangiomas to identify radiological features specific to vascular bone tumors. Aggressive features, including soft tissue components, cortical expansion, and destruction, were identified in four cases, raising suspicion for malignancy. Eight cases demonstrated lobulated lytic lesions with sclerotic margins, and four cases showed trabeculation, leading the authors to conclude that histology remains the only reliable diagnostic method [2]. Vermaat et al., focusing on the radiology of malignant vascular bone tumors, described predominantly well-demarcated lytic lesions, with 58% of cases showing reactive changes in bone and soft tissue. They suggested that these features might aid differential diagnosis by distinguishing these tumors from metastases. However, similar reactive changes may also occur in benign vascular tumors, as observed in our series [5].

In our cohort, hemangiomas generally exhibited mild radiological features, whereas epithelioid hemangiomas, classified as locally aggressive, presented with larger tumor size in both cases; cortical expansion, destruction, and contrast enhancement were present, and one lesion had both a soft tissue component and a pathological fracture. High-grade angiosarcomas exhibited aggressive radiological characteristics, typically as rapidly enlarging, large lesions with soft tissue components and pathological fractures. These findings are consistent with previously reported literature [11, 12].

Hemangiomas are benign, and symptomatic treatment yields excellent survival, whereas epithelioid hemangiomas are considered intermediate-grade tumors, with a 2% risk of metastasis and a 9% recurrence rate; treatment with curettage or marginal excision is usually sufficient [7]. In our study, both hemangiomas and epithelioid hemangiomas showed excellent outcomes. All angiosarcoma cases in our series resulted in death due to disease or complications, reflecting a very poor prognosis. The low-grade epithelioid hemangioendothelioma case in our series has remained under follow-up for approximately 10 years despite pulmonary metastases. Epithelioid hemangioendothelioma is known to have an indolent course with a 20–30% risk of metastasis and a 15% mortality rate [1].

Our findings underscore the dramatic differences in prognosis between benign and malignant vascular bone tumors. While benign lesions in our series exhibited no complications or recurrences, malignant tumors demonstrated high rates of relapse and mortality. The literature supports these results, showing that intralesional curettage or marginal excision yields excellent outcomes in benign hemangiomas and epithelioid hemangiomas [1]. In contrast, angiosarcomas are characterized by rapid progression, high recurrence risk, and a predilection for complex anatomical regions such as the pelvis, which often results in delayed diagnosis, all contributing to poor prognosis. Previous reports corroborate the poor outcomes of high-grade angiosarcoma: Evans et al. described three bone angiosarcoma cases resulting in death from metastases at 3 months, 13 months, and 7 years despite treatment [13], while Righi et al. reported that 37 of 45 patients with epithelioid angiosarcoma died during short-term follow-up (median 22 months), with a mortality rate of 73% and mean survival of 10 months [6]. Although (neo-)adjuvant chemotherapy and radiotherapy may improve outcomes in unresectable angiosarcomas, no specific chemotherapeutic agent has proven efficacy, and even in cases of resectable localized tumors, metastatic relapse occurs in approximately 50% of patients, resulting in low survival [1].

This study has several limitations. Due to the rarity of these tumors and their unusual anatomical locations, the sample size was relatively small. Additionally, the analysis was retrospective. Nonetheless, the ability to perform a comparative analysis is a strength of this study. Radiological images and pathology slides were re-evaluated to ensure diagnostic accuracy, and definitive diagnoses were assigned for all cases. However, given the small sample size and the rarity of primary vascular bone tumors, the statistical analyses should be interpreted with caution. All analyses were exploratory in nature and intended to generate hypotheses rather than establish definitive diagnostic criteria.

## Conclusion

In conclusion, the presence of a soft tissue component and larger tumor size were the most useful radiological features associated with malignancy in vascular bone tumors of the pelvis and extremities. In this cohort, tumor size around 5.5 cm was associated with malignancy and may serve as an exploratory imaging indicator; however, it should not be interpreted as a definitive diagnostic threshold. Pathological fractures and cortical destruction may also occur in benign lesions and therefore cannot be used independently for differential diagnosis. Benign tumors generally yield excellent outcomes with curettage, whereas malignant tumors are associated with high rates of recurrence and mortality. Consequently, imaging findings must be corroborated with histopathological confirmation for definitive diagnosis. Given the rarity of these tumors, larger series are needed to validate these findings and further refine diagnostic and prognostic criteria.

## Data Availability

No datasets were generated or analysed during the current study.
